# Flexible growth and body mass predict physiological condition at fledging in the synchronously breeding European starling, *Sturnus vulgaris*

**DOI:** 10.1098/rsos.220583

**Published:** 2022-06-08

**Authors:** Joshua M. Allen, Brett L. Hodinka, Hannah M. Hall, Kathryn M. Leonard, Tony D. Williams

**Affiliations:** Department of Biological Sciences, Simon Fraser University, Burnaby, British Columbia, Canada

**Keywords:** pre-fledging mass recession, compensatory growth, aerobic capacity, oxidative status, developmental plasticity

## Abstract

Recent studies have reported beneficial carryover effects of juvenile development that predict interspecific survival differences at independence. Yet, traits relating to body size (i.e. morphological traits) have proven to be unreliable predictors of juvenile survival within species. Exploring individual variation of growth trajectories and how they covary with physiology could reveal species-specific developmental modes which have implications for our assessments of juvenile quality. Here, we investigated morphological development of European starlings (*Sturnus vulgaris*) approaching fledging in relation to three components of physiological condition at independence: aerobic capacity, energy state and oxidative status. We found evidence of flexible mass and wing growth which independently covaried with fledgling energy state and aerobic capacity, respectively. By comparison, tarsus and wing length at fledging were unrelated to any physiological trait, while mass was positively associated with principal component scores that comprised aerobic capacity and energy state. Thus, flexible growth trajectories were consistent with ‘developmental plasticity’: adaptive pre-fledging mass recession and compensatory wing growth, which seemingly came at a physiological cost, while fledgling body mass positively reflected overall physiological condition. This highlights how patterns of growth and absolute size may differently reflect fledgling physiology, potentially leading to variable relationships between morphological traits and juvenile fitness.

## 1. Introduction

Ontogenetic growth and fitness are intimately related due to strong effects of body size on fecundity and longevity [1]. In birds and mammals, size at maturity is a key determinant of future survival (reviewed in [2]) and predicts that there should be strong selection to prioritize growth or developmental maturity of any trait that contributes to fitness [3–7]. In birds, differences in developmental maturity at fledging (nest departure) have been used to explain variation in post-fledging mortality across taxa [7–9]. However, our understanding of developmental maturity in birds is often limited to measures of absolute body size, typically mass and/or structural size (e.g. wing length), restricting our understanding of individual quality (*sensu* [10]) to predominantly morphological traits (i.e. relating to chick size). Morphological traits have proven useful in understanding variation in rates of juvenile mortality between species [7,9], but have produced mixed results within species [2,7,8,11,12].

To address the equivocal nature of studies to date, it is important to consider causative versus correlative links between morphological traits and survival. Increased wing length may be causally related to decreased fledgling mortality because it improves flight ability [7,13], likely facilitating the evasion of predators [14]. However, some morphological traits (e.g. body mass) are supposedly correlated with survival because they reflect physiological ‘condition’ or quality [15–17]. Yet, empirical evidence linking morphological and physiological traits in juveniles is lacking, especially considering the variability of developmental trajectories between species. For example, many species display mass-overshoot-recession growth profiles where asymptotic mass is attained well before fledging, followed by rapid pre-fledging mass recession [18–23]. Furthermore, while some morphological traits such as tarsus and wing length generally increase linearly with age, final size at fledging can be influenced by earlier developmental conditions and compensatory growth [24–28]. In such cases, it is uncertain how varying morphological trajectories interact with a suite of physiological traits (e.g. through developmental trade-offs) that have themselves also been linked to fitness [6,13,29–32], especially approaching fledging. Considering fledging represents a key life-stage transition marked by high levels of mortality [33], investigating relationships among traits at this transition could provide insights into how variable developmental trajectories (cf. absolute size) facilitate carryover effects between life-stages to influence overall fitness.

In this study, we measured developmental trajectories of morphological traits (mass, tarsus and wing length) approaching independence and final physiological condition (aerobic capacity, energy state and oxidative status) in the European starling (*Sturnus vulgaris*), a species that displays a mass-overshoot-recession growth profile [34]. We analysed changes in morphological traits approaching fledging in relation to physiological traits at fledging, while also assessing relationships among traits at fledging. Our objectives were to (i) examine individual variation in the developmental trajectories of morphological traits approaching fledging, exploring patterns that may reflect developmental plasticity (e.g. adaptive mass recession), (ii) assess if the individual variation of morphological developmental trajectories predict fledgling physiology, consistent with developmental trade-offs and/or preferential resource allocation and (iii) investigate relationships among morphological and physiological traits at fledging, testing the assumption that traits relating to body size are useful metrics of overall quality because they reflect physiological condition.

## 2. Methods

In 2020 and 2021, we measured morphological developmental trajectories approaching fledging and overall morphological and physiological condition at fledging in European starlings at our long-term study side of Davistead Farm, Langley, British Columbia, Canada (49°10′ N, 122°50′ W). Here, nest-boxes are monitored daily from egg-laying until fledging or failure to determine lay date, egg size and clutch size. At 15 days post-hatching, when European starlings typically reach asymptotic mass, two chicks were randomly selected from broods of 4–6 chicks (mean brood size 17 days after hatching = 4) as controls for a separate experiment. These control chicks provided all the data presented within this study.

### 2.1. Field and laboratory methods

Chicks (*n* = 98) at first brood nests (*n* = 49) were weighed (body mass, ± 0.01 g) and measured (tarsus, ± 0.01 mm, wing chord, ± 0.5 mm) 15, 17 and 20 days after hatching between 10.00 and 16.30 to assess morphological development. Also on day 20, one day prior to typical fledging age (greater than 90% of chicks fledge on day 21; [13]), we used heparinized capillary tubes to take 100–500 µl of blood (less than 10% of total blood volume) from the wing's brachial vein, immediately following initial nest disturbance to minimize handling time. Handling times were calculated for each individual and were defined as the time between initial nest disturbance and the time each chick was weighed (for days 15–20) or blood sampled (day 20). Mass was not related to handling time at any age (*p* > 0.22 in all cases) nor time of day (*p* > 0.38). Additionally, no physiological trait showed any relation to handling time (*p* > 0.36) or time of day (*p* > 0.22). Using these samples (*n* = 89), we assessed fledgling physiological condition by measuring three distinct traits: aerobic capacity (haematocrit and haemoglobin), energy state (plasma triglycerides) and oxidative status (reactive oxygen metabolites (dROMs) and antioxidant titers (OXY)). Nest-boxes were checked at day 22 to confirm fledge success; nine chicks fledged before day 20 measurements and all remaining chicks fledged by day 22. Birds that fledged before day 20 were not blood sampled and therefore not included in any analysis of physiological traits.

Haematocrit (% packed cell volume) was measured (± 0.01 mm) using digital calipers after whole blood was centrifuged for 3 min at 13 000 g (Microspin 24; Vulcon Technologies, Grandview, MO, USA). Haemoglobin (g dl^−1^) was measured using the cyanomethaemoglobin method [35] modified for use with a microplate spectrophotometer (BioTek Powerwave 340; BioTek Instruments, Winooski, VT, USA), measuring 5 µl whole blood diluted in 1.25 ml Drabkin's reagent (D5941; SigmaAldrich Canada, Oakville, Ontario, Canada) at 540 nm absorbance. Plasma triglycerides (mmol l^−1^) were measured with a colorimetric assay (Sigma-Aldrich Co.) following the manufacturer's guidelines. Reactive oxygen metabolites (mg H_2_O_2_ dl^−1^) and total antioxidant titers (µmol HClO ml^−1^) were measured using dROMs and OXY kits from Diacron International (Grosseto, Tuscany, Italy). Using a single pooled plasma sample, we calculated intra-assay coefficients of variation (CV) by running 10 replicates of this pool on an initial quality control plate (haemoglobin = 0.9%; plasma triglycerides = 2.67%; OXY = 9.92%; dROMs = 4.77%). Each subsequent plate was run alongside the same pool to calculate inter-assay CVs (haemoglobin = 2.77%; plasma triglycerides = 6.06%; OXY = 4.27%; dROMs = 5.98%). Samples were run in duplicate or triplicate; any sample with a CV greater than 10% was rerun. Red blood cells (*n* = 43) were sent to HealthGene Laboratory (Concord, Ontario, Canada) for sexing by polymerase chain reaction alongside known adult samples for quality control. Sex was only known for one year due to a freezer malfunction (during the COVID-19 lockdown) that resulted in the loss of red blood cells from 2020.

### 2.2. Statistical methods

Statistical analyses were performed in RStudio v. 4.0.3 (RStudio, *Inc.*, Boston, MA, USA). We tested all traits for normality (Shapiro–wilks test); plasma triglycerides and dROMs data were logarithmically transformed. Tarsus and wing length were not normally distributed but showed no improvement when transformed. We therefore used quantile–quantile plots of studentized residuals for models involving tarsus and wing length to remove points of undue influence which significantly deviated (fell outside 95% confidence bands) from the normal distribution; a maximum of four individuals were removed from any single model. Akaike's information criterion (AIC) was used to select main effect-covariate interaction terms. Interaction terms were incorporated where a lower AIC score supported their inclusion and are reported when significant. In-text values are presented as means ± s.d.

We first ran bivariate analyses to independently test the effects of ecological context (brood size and year) and sex on pre-fledging morphological development and fledgling (day 20) morphological and physiological maturity. We created linear mixed-effect models (LMMs) using the change (Δ) in morphological traits (mass or wing length) between ages 15 and 20 and ecological context and sex as main effects. Nest ID was included as a random factor in all our models. Since chick tarsi were fully developed by 15 days old (mean Δ tarsus length between day 15 and 20 = 0.01 ± 0.39 mm), displaying no relationship with age (LMM: *p* = 0.38), we only considered tarsus length at day 20 in our models and did not analyse Δ tarsus length. We used similar LMMs to test for ecological context and sex effects on fledgling developmental maturity, replacing Δ morphological traits with day 20 morphological and physiological trait measurements as main effects. Next, we tested for relationships among morphological and physiological traits using LMMs and controlling for ecological context and sex. Results from models testing for effects of ecological context and sex on development, as well as covariation among morphological and physiological traits, are reported in the electronic supplementary material. These models informed covariate selection within our subsequent analyses; we included any significant effects (i.e. *p* < 0.05) as covariates. Where sex was used as a covariate, models were first run without sex as a covariate and using both years of data, then again using only 2021 data to test for covariance by sex.

**Figure 3.**
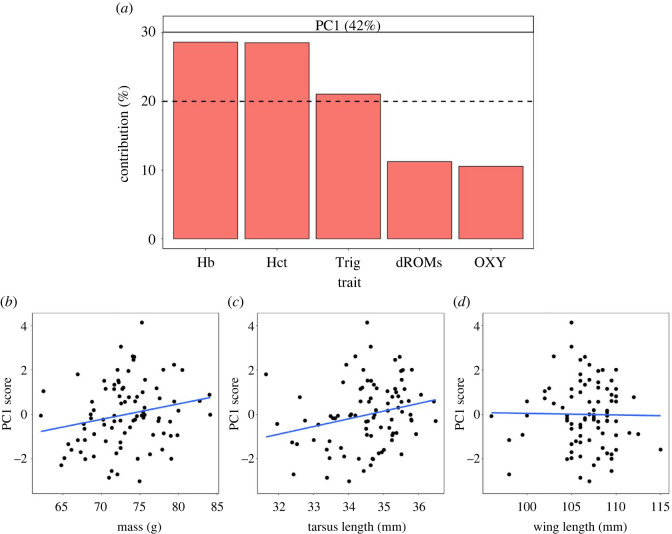
PCs of fledgling physiology versus morphological traits. (*a*) Contributed variance of physiological traits to PC one (PC1) of fledgling physiology. Individual scores for PC1 in relation to day 20 (*b*) mass, (*c*) tarsus length and (*d*) wing length. Dashed line in (*a*) is the expected average contributed variance by each trait to the PC. Only the relationship in (*b*) was significant (see ‘Results’). Hb, haemoglobin; Hct, haematocrit; Trig, plasma triglycerides; dROMs, reactive oxygen metabolites; OXY, antioxidant titers.

To explore morphological development prior to fledging, we tested whether morphological traits at day 15 predicted subsequent changes to day 20 by using day 15 mass or wing length and Δ mass or wing length between days 15 and 20 as main effects. We used LMMs with repeated measures to test for relationships between morphological traits and age (days 15–20), using chick ID nested within nest ID as random factors. Finally, we used two approaches to investigate associations between morphological development and fledgling physiology: (i) LMMs to analyse direct correlations between individual morphological and physiological traits, potentially reflecting mechanistic links between growth and physiology, and (ii) principal component (PC) analysis with LMMs to test whether morphological traits at fledging predict overall indices of physiological condition. For the first approach, we tested whether each of the five physiological traits at day 20 was independently correlated with Δ morphological traits (mass and wing length) between ages 15 and 20 and day 20 measurements (mass, tarsus length and wing length). For the latter approach, we identified only one principal component (PC1) of physiological condition with an eigenvalue significantly greater than 1 (PC1 eigenvalue = 2.1), which explained 42% of the variance in fledgling physiology. Three of the five physiological traits contributed to the majority of PC1 variance (figure 3*a*): haemoglobin (loading: 0.54), haematocrit (0.53) and plasma triglycerides (0.46), each of which was significantly positively correlated with PC1 (*p* < 0.01 in all cases). We calculated PC scores for each individual for PC1 and tested for general associations between individual variation in these PC scores and morphological measurements at day 20, using LMMs with nest ID nested within year as random factors.

## 3. Results

### 3.1. Individual variation in the developmental trajectories of morphological traits

**Figure 1.**
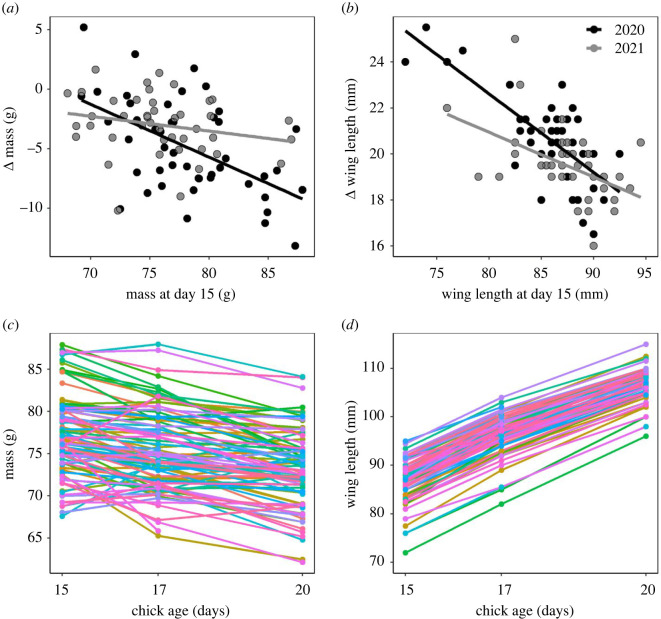
Individual variation of morphological development approaching fledging in the European starling (*Sturnus vulgaris*) across two years. (*a*) Mass and (*b*) wing length 15 days after hatching in relation to subsequent changes (Δ) in these traits between days 15 and 20. (*c*) Mass and (*d*) wing length on days 15, 17 and 20. All relationships were significant and are reported in ‘Results’. For covariation of morphological development by year, see electronic supplementary material, table S1.

Mass at day 15 was significantly negatively correlated with Δ mass to day 20 (figure 1*a*; *p* < 0.01, *F*_1,80_ = 11.3), and there was a significant mass × brood size interaction (*p* < 0.01, *F*_1,79_ = 7.28), mass × year (*p* = 0.02, *F*_1,78_ = 5.81), year × brood size (*p* = 0.04, *F*_1,77_ = 4.49) and mass × brood size × year interaction (*p* = 0.04, *F*_1,77_ = 4.44). The brood size × year interaction revealed a positive, but non-significant, relationship between brood size and mass loss in 2020 (*p* = 0.24, *F*_1,22_ = 1.48) but a negative relationship in 2021 (*p* = 0.09, *F*_1,15_ = 3.34). In 2020, brood sizes of four displayed the greatest Δ mass between days 15 and 20 (−7.27 ± 5.14 g), whereas in 2021, Δ mass was greatest in brood sizes of six (−3.68 ± 2.5 g). Despite a significant mass × year interaction, the negative relationship between mass at day 15 and Δ mass between days 15 and 20 was significant in both 2020 (*p* < 0.01, *F*_1,42_ = 13.9) and 2021 (*p* = 0.02, *F*_1,31_ = 5.69). Day 15 wing length was also significantly negatively correlated with subsequent Δ wing length to day 20 (figure 1*b*; *p* < 0.01, *F*_1,86_ = 63.5).

Mass was significantly negatively related to age between days 15 and 20 (figure 1*c*; *p* < 0.01, *F*_1,187_ = 155), with chicks losing 3.98 ± 3.48 g between days 15 and 20. However, wing length was significantly positively related to age (figure 1*d*; *p* < 0.01, *F*_1,185_ = 10 195), growing 20 ± 1.84 mm between days 15 and 20. To test if the negative correlations between size at day 15 and subsequent changes to day 20 reduced individual variation in morphological traits approaching fledging, we performed post hoc *F*-tests to compare the variance of morphological traits at day 15 versus day 20. There was no significant difference in the variance of mass between days 15 and 20 in 2020 (*p* = 0.55, *F*_1,45_ = 1.2) or 2021 (*p* = 0.81, *F*_1,42_ = 0.93). However, variance in wing length at day 20 was significantly lower than at day 15 (*p* = 0.04, *F*_1,87_ = 1.55).

### 3.2. Pre-fledging morphological growth trajectories in relation to fledgling physiology

**Figure 2.**
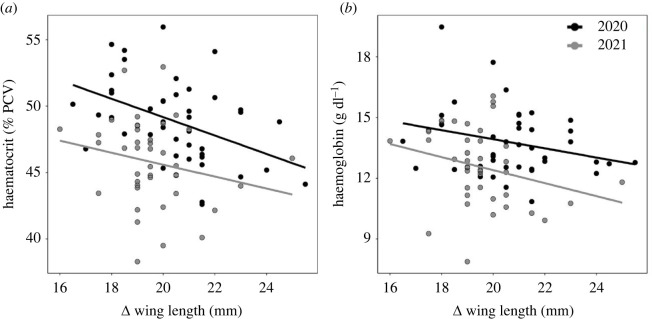
Covariation between wing growth approaching fledging and fledgling aerobic capacity. Change (Δ) in wing length between days 15 and 20 (*a*,*b*) in relation to day 20 (*a*) haematocrit and (*b*) haemoglobin. Both relationships were significant (see ‘Results’). For covariation of physiological traits by year, see electronic supplementary material, table S1.

Individual variation in Δ mass between days 15 and 20 was significantly positively related to day 20 plasma triglycerides (*p* < 0.01, *F*_1,75_ = 7.36) but showed no relation to haematocrit (*p* = 0.72, *F*_1,79_ = 0.13), haemoglobin (*p* = 0.36, *F*_1,79_ = 0.86), dROMs (*p* = 0.13, *F*_1,80_ = 2.3) or OXY (*p* = 0.16, *F*_1,78_ = 1.99). Individual variation in Δ wing length between days 15 and 20 was significantly negatively related to day 20 haematocrit (figure 2*a*; *p* = 0.03, *F*_1,76_ = 5.11) and haemoglobin (figure 2*b*; *p* < 0.01, *F*_1,66_ = 8.64), but showed no relationship to plasma triglycerides (*p* = 0.07, *F*_1,79_ = 3.37), dROMs (*p* = 0.73, *F*_1,81_ = 0.12) or OXY (*p* = 0.66, *F*_1,80_ = 0.2).

### 3.3. Relationships between morphological and physiological traits at fledging

There was a positive correlation between day 20 mass and plasma triglycerides that approached significance (*p* = 0.05, *F*_1,74_ = 3.83); however, this relationship was only significant in 2021 when we could control for sex (*p* = 0.04, *F*_1,32_ = 4.72). Haematocrit, haemoglobin, dROMs and OXY were all independent of day 20 mass (*p* > 0.09 in all cases). However, mass was significantly positively related to PC1 (figure 3*b*; *p* = 0.04, *F*_1,84_ = 4.19). Tarsus and wing length at day 20 were unrelated to all physiological traits (*p* > 0.3 in all cases), as well as PC1 (figure 3*c,d*; *p* > 0.76 in all cases).

## 4. Discussion

We investigated covariation between morphological and physiological development approaching fledging in juvenile European starlings, focusing on the changes of morphological traits just prior to fledging and correlating these to physiological traits at fledging. Individual variation in *changes* of morphological traits were consistent with marked development plasticity and were better predictors of individual physiological traits at fledging than absolute fledgling size. Nestling mass and wing length at day 15 strongly predicted subsequent growth to day 20, suggesting adaptive mass recession and compensatory wing growth, respectively, which coincided with a significant decrease in the variance of wing length over the same period. Such plasticity may function to ensure beneficial carryover effects into the post-fledging period, as flexibility of these traits may promote juvenile flight ability (e.g. optimizing wing load; [13,22,23]), a strong predictor of survival between bird species [7,9]. However, our data suggest physiological costs for flexible growth, potentially associated with resource allocation decisions, as increased mass recession and wing growth approaching fledging were associated with decreased energy state and aerobic capacity. This covariation could lead to similar fitness-related morphological traits at fledging (e.g. wing length) but variable and hidden physiological costs, paradoxically reducing the potential for such traits to predict post-fledging survival [5]. Furthermore, we identified one PC of fledgling physiology, explaining 42% of physiological variance across individuals which represents the best metric of overall physiological condition. Fledgling mass was positively correlated with PC1; however, wing and tarsus length at fledging were independent of all physiological traits measured, as well as PC1. This suggests that metrics of both growth and body size may reflect physiological condition, but these relationships may vary between morphological traits and depend on whether physiological traits are considered individually (as in many studies) or collectively.

### 4.1. Individual variation in the developmental trajectories of morphological traits

The development of morphological traits approaching fledging has previously been reported in this species, revealing significant wing growth but mass recession [34], as seen here. However, by exploring these trajectories further, we have shown that morphological trait values at 15 days after hatching strongly predict subsequent growth to fledging at day 20. Pre-fledging mass recession was highly variable among individuals and was seemingly dictated by asymptotic mass; heavier individuals at day 15 generally lost more mass to day 20, whereas some individuals showed no change in mass or even an increase in mass between days 15 and 20. This may also explain the variation of mass trajectories by year (electronic supplementary material, table S1), as chicks in 2020 were significantly heavier on day 15 but lost more mass to day 20. Consequently, chick mass on day 20 was similar between years (electronic supplementary material, table S1), despite very different pre-fledging trajectories. Thus, pre-fledging mass recession does not follow a hardwired developmental trajectory in European starlings but is instead a flexible process that can be adjusted based on the rate of mass gain prior to asymptotic mass, which likely varies by ecological context. This is consistent with experimental studies which have demonstrated facultative mass recession in swifts [22,23], but are somewhat contradicted by similar studies in other species with mass-overshoot-recession growth profiles where nestlings showed no adjustment to an experimental increase in their pre-fledging mass [36,37].

In addition to developmental plasticity of mass trajectories approaching fledging, nestlings displayed flexible wing growth between days 15 and 20 in relation to their wing length at day 15. Nestlings with smaller wings at day 15 generally grew their wings at a much greater rate to day 20 than individuals with longer wings. This pattern is consistent with compensatory growth, in which animals exhibit faster than usual growth rates following a period of resource deprivation [38]. Consequently, variance in nestling wing length decreased as wing lengths converged towards a mean value at fledging. Fledging with underdeveloped wings likely incurs higher post-fledging mortality [7,9]. However, instead of delaying fledging to facilitate catch-up growth [24,39,40], European starlings seemingly prioritize accelerated, compensatory growth approaching independence, perhaps to facilitate synchronous fledging within and/or across broods. This supports the idea that compensatory growth evolves where there are constraints on development time [41,42].

While compensatory growth is typically defined by a return to more favourable conditions following a period of fasting [43], we do not have the data to support this mechanism. While we have several years of data suggesting there is no change in provisioning rate by nestling age in European starlings [44], we cannot rule out that changes in food allocation by parents prior to fledging (perhaps induced by increased begging in smaller nestlings; [45]) is the mechanism for compensatory growth in this species. However, we suggest that an alternative mechanism for compensatory growth could be preferential resource allocation towards (or developmental trade-offs favouring) key fitness traits approaching life-stage transitions (see below), that need not be facilitated by realimentation [46,47].

Decreased individual variation of wing length approaching fledging is consistent with developmental canalization, whereby traits with larger fitness effects (like wing length: [7,9,13,48]) exhibit less phenotypic variation in response to genetic and environmental changes [3,5,6,49]. Indeed, wing growth was seemingly robust to contextual perturbations, developing independently of brood size, sex and year (electronic supplementary material, table S1). As such, trajectories of structural traits may appear to be developmentally hardwired when observed as an average across a population and/or species. However, by exploring variable growth between individuals, we have demonstrated a high level of flexibility of wing development which suggests certain traits may be more plastic than previously thought in some species. Such plasticity does not negate canalized wing development though, as variable growth rates resulting from preferential resource allocation (reviewed in [50]) may in fact promote an ecological robustness of certain traits [51].

### 4.2. Pre-fledging morphological growth trajectories in relation to fledgling physiology

There was covariation between morphological and physiological traits approaching fledging, suggesting potential energetic costs of pre-fledging mass recession and aerobic costs of compensatory wing growth. Decreased plasma triglyceride levels in relation to greater mass recession is consistent with a mechanism of lipid catabolism underpinning pre-fledging mass recession [52–54], potentially contradicting Lack's [55] energy reserves hypothesis suggesting fitness advantages for fatter fledglings (but see below). Furthermore, there is some support to suggest nestlings are not losing mass solely by fasting, as this may lead to a reduction in metabolites and protein oxidation [56], while we found no relationship between mass recession and indices of oxidative status here. This is consistent with previous studies showing no change in provisioning rate or begging intensity during pre-fledging mass recession [23,37].

Flexibility of wing development was also associated with physiological costs, as compensatory wing growth seemingly resulted in decreased fledgling aerobic capacity. Similar results have been reported in laboratory studies showing nestlings directing investment towards structural growth at the expense of organ development in response to food shortages [57,58]. Studies that have explored physiology in relation to compensatory growth largely focus on oxidative and metabolic costs [27,59–64], yet few have tested immediate trade-offs between such growth trajectories and other metrics of physiological quality, such as aerobic capacity and energy state. These immediate trade-offs are potentially more significant determinants of recruitment than deferred costs, as they are more pertinent to the immediate post-fledging period and the high rates of juvenile mortality that characterize it [33]. While many studies have shown oxidative costs associated with growth (reviewed in [61]), we found no evidence to support that here. Perhaps it is plausible that individuals transitioning to independence with reduced aerobic capacity, a trait linked to post-fledging flight ability and survival [7,29,31,32], experience increased oxidative stress later in life as the physiological immaturity of these traits fail to meet performance demands.

### 4.3. Relationships between morphological and physiological traits at fledging

Much work has explored whether individual variation in morphological traits reflect physiological quality but mainly in mature, adult animals [15,17,65–67]. However, in nestlings where both suites of traits are still developing, it is uncertain how morphological and physiological conditions are related. Despite this, morphological traits alone are often used to assess individual quality at fledging, often under the assumption that variation in these traits reflects physiological ‘quality’. We found evidence to suggest body mass, but not structural size, at independence reflects physiological condition. Despite marked mass recession prior to fledging, fledgling mass was nonetheless positively associated with PC1. The majority of the variance in PC1 was explained by metrics of aerobic capacity, which have been linked to flight ability in European starlings previously [13]. Thus, the contribution of aerobic traits towards flight ability may enable individuals of better physiological quality to maintain more mass prior to fledging, potentially to experience adaptive benefits of improved energy state associated with increased mass shown here. Conversely, the lack of an association between structural traits and physiological condition may be driven by plasticity of growth trajectories prior to fledging, which reflected individual variation of fledgling aerobic capacity. While this plasticity may facilitate the attainment of normal fledgling size, it potentially leads to greater physiological variation as observed previously [34]. The resulting decreased variance in key fitness traits (e.g. wing length) approaching fledging potentially uncouples any correlations among developing structural and physiological traits.

The ecological robustness and developmental prioritization of wing growth approaching fledging supports recent studies suggesting wing size is a morphological trait with strong links to post-fledging survival between species [7,9], providing further evidence that this trait is canalized [5]. However, such canalization may actually reduce the potential for wing length to predict post-fledging survival within species, as observed in [7]. This may be due to weak links with developmental conditions [5] and/or trade-offs that facilitate canalized morphological developmental trajectories at the expense of physiological condition ([57,58]; this study). Thus, body mass and wing length may be variously linked to rates of juvenile mortality through indirect versus direct associations with fitness; mass may generally reflect physiological traits that are correlated with survival, whereas wing length may directly determine survival by reflecting juvenile flight ability, but inconsistencies at predicting survival within species could be related to developmental trade-offs between growth and physiology, as suggested here.

## Data Availability

The data are provided in the electronic supplementary material [68].
